# Survival capability of *Campylobacter upsaliensis* under environmental stresses

**DOI:** 10.1186/s13104-022-05919-2

**Published:** 2022-02-14

**Authors:** Walid Elmonir, Torrung Vetchapitak, Tomoko Amano, Takako Taniguchi, Naoaki Misawa

**Affiliations:** 1 Hygiene and Preventive Medicine (Zoonoses) Department, Faculty of Veterinary Medicine, Kafrelsheikh University, Kafrelsheikh, 33511 Egypt; 2 Center for Animal Disease Control, University of Miyazaki, 1-1 Gakuenkibanadai-nishi, Miyazaki, 889-2192 Japan; 3 Laboratory of Veterinary Public Health, Department of Veterinary Medical Science, Faculty of Agriculture, University of Miyazaki, 1-1 Gakuenkibanadai-nishi, Miyazaki, 889-2192 Japan

**Keywords:** *Campylobacter upsaliensis*, Environmental stress, Survival, *Campylobacter jejuni*, Dog food, Dog feces

## Abstract

**Objective:**

*Campylobacter upsaliensis* has been recognized as an emerging pathogen. However, little is known about its survival in the environment. To evaluate its survival capability, we estimated the reduction in viable counts of *C. upsaliensis* after aerobic exposure to starvation in phosphate-buffered saline (PBS), acidity (pH = 4.3), high osmolarity (4% NaCl), and dryness in wet pulp disks at different temperatures. Also, survival in dog feces and dog food at variable temperate was assessed.

**Results:**

*Campylobacter upsaliensis* remained culturable under starvation for 4 days at 25 °C and for 10 weeks at 4 °C. *C. upsaliensis* was also recoverable after exposure to high osmolality for 9 days, dryness for 5 days, and acidity for 2 days, respectively. Similarly, *C. upsaliensis* survived in dog feces and dog food for several days at 25 °C and weeks at 4 °C. The survival capability of the organism was dependent on the water content, and also temperature. Notably, the tested *C. upsaliensis* strain was less resilient under all tested conditions than a *C. jejuni* strain used as a control. The findings showed that *C. upsaliensis* is able to survive under various environmental stresses, suggesting that it could pose a potential threat to public health.

## Introduction

*Campylobacter upsaliensis* (Cups), the most common *Campylobacter* species found particularly in dogs, has been recognized as an emerging human pathogen [1]. The Cups infection is associated with a self-limiting diarrheal disease in most human cases; other serious conditions have also been reported, including bloody diarrhea, bacteremia, Guillain-Barré syndrome and hemolytic-uremic syndrome [2, 3]. Epidemiological studies have indicated that not only indirect transmission such as foodborne infection but also direct contact with infected dogs poses a significant risk for Cups infection in humans [3]. Children are thought to be more vulnerable to this risk. In fact, a significant association between cases of the infection in the ≤ 5-year age group and the presence of a puppy in the household has been demonstrated [4, 5].

Although *Campylobacter jejuni* (Cj) requires microaerophilic conditions, this pathogen has acquired the ability to adapt to severe environmental conditions to maintain its life cycle [6]. However, little is known about the survival capabilities of Cups in the environment.

The present study was performed to evaluate the survival of Cups under various environmental stresses. We examined the stress responses of Cups to starvation, dryness, acidity, and osmolality in an aerobic atmosphere at 4 °C and 25 °C. We also examined its survival in dog feces and dog food at both ambient temperatures.

## Main text

### Materials and methods

#### Bacterial strains

Cups LMG8850 was obtained from the Belgian Co-ordinated Collections of Micro-organisms (BCCM), Belgium. A human clinical strain, Cj 81-176, was used as a control [7]. Both strains were grown on blood agar No. 2 (Kanto Kagaku, Tokyo, Japan) containing 5% defibrinated horse blood (Nippon Biotest Laboratories, Tokyo, Japan) at 37 °C for 48 h under microaerobic conditions (75% N_2_, 10% CO_2_, 5% H_2_, and 10% O_2_).

#### Survival of *Campylobacter* species exposed to starvation, dryness, acidity and high osmolality

##### Survival to starvation

The 48-h cultivated Cups and Cj cells on blood agar plates were harvested in 10 mM phosphate-buffered saline (PBS, pH 7.2) and the optical density at 550 nm (OD_550_) was adjusted to 0.1, representing 8 log_10_ colony-forming unit (CFU)/ml. Aliquots of bacterial suspension (1 ml) were dispensed into glass tubes and covered with an aluminum cap. They were cultured aerobically in incubators at 25 °C or 4 °C. After predetermined incubation periods, the number of CFU in the PBS was measured by direct plating on blood agar.

##### Survival to dryness

Aliquots of 50 μl of bacterial suspension (0.1 OD_550_) were dropped onto sterilized pulp disks 10 mm in diameter used for detection of antimicrobial agents (Toyo Roshi, Tokyo, Japan). These were placed in covered petri dishes 90 mm in diameter (Iwaki, Tokyo, Japan) and incubated aerobically at 25 °C or 4 °C. After predetermined incubation periods, the weight variation of the pulp disks was measured, and then the number of CFU in the disk was determined by direct plating on blood agar.

Survival to acidic or high osmolality stress was conducted as described elsewhere [7]*,* with some modifications. In brief, the pH of Brucella broth (Beckton Dickinson, MD, USA) was adjusted to 4.3 by adding hydrochloric acid (HCl) (Nacalai Tesque, Kyoto, Japan) and used as a form of acidic stress. For the high osmolality stress test, 3.5 g of sodium chloride (NaCl) (Nacalai Tesque) was added to 100 ml of Brucella broth resulting in a final concentration of 4% NaCl, since Brucella broth already contains 0.5% NaCl. The 48-h cultivated *Campylobacter* spp. were inoculated in the modified Brucella broths, and the OD_550_ of both inoculated broths was adjusted to 0.1. The bacterial counts were conducted as described above.

#### Bacterial survival in dog feces, and dog food artificially spiked with *Campylobacter* spp.

##### Survival in dog feces

Stool material was collected from a healthy adult dog and confirmed to be *Campylobacter* spp.-negative using enrichment followed by direct plating culture [8]. This fecal material (20 g) was then inoculated by mixing 20 ml of 8 log_10_ CFU/ml Cups and Cj in a sterile stomacher bag (Central Scientific Commerce Inc., Tokyo, Japan), and homogenized for 2 min. Then, 1-g aliquots of feces were placed in covered petri dishes 35 mm in diameter (Iwaki) and incubated aerobically at 25 °C or 4 °C. After predetermined incubation periods, the number of culturable cells in the fecal sample was measured by direct plating on Skirrow selective agar plates (Kanto Kagaku) after 4 days of incubation at 37 °C under microaerobic conditions as described above.

##### Survival in dog food

A wet-type dog food (water content 80%) and a dry-type dog food (water content 10%) whose major ingredients were chicken meat and beef were purchased from a pet shop. Each food (20 g) was inoculated with 20 ml of 8 log_10_ CFU/ml bacterial cells in a sterile stomacher bag. The wet-type food was then homogenized for 2 min but the dry type was suspended for 10 min before homogenization. The incubation, the food weight measurement, and the CFU count were conducted as described above.

#### Statistical analysis

Each experiment was repeated three times and mean microbial counts were converted to log_10_ CFU/g. Pearson’s correlation coefficient (R) was used to examine the relation between Cups and Cj counts and the respective weights of feces, food and wet pulps. Statistical significance was defined as *P* ≤ 0.05.

### Results and discussion

#### Survival of campylobacters in PBS under an aerobic atmosphere

**Fig. 1:**
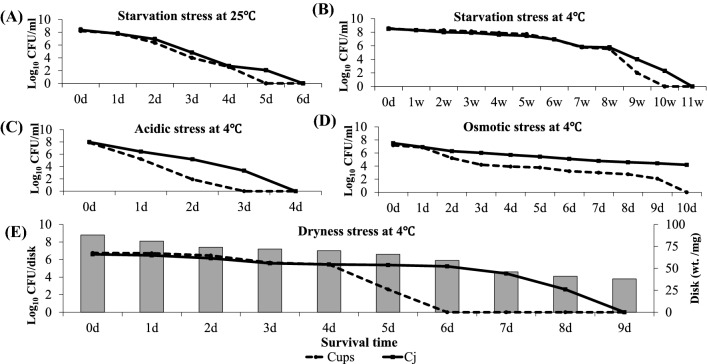
Survival of *Campylobacter* spp. exposed to starvation, dryness, acidity and osmolarity stress in aerobic atmosphere at different temperatures. **A** Effect of starvation on the survival of *C. upsaliensis* (Cups) and *C. jejuni* (Cj) at 25 °C. **B** Effect of starvation on the survival of Cups and Cj at 4 °C. **C** Effect of acidic stress on the survival of Cups and Cj at 4 °C. **D** Effect of osmotic stress on the survival of Cups and Cj at 4 °C. **E** Effect of dryness stress on the survival of Cups and Cj at 4 °C. Survival capability was determined in terms of log_10_ culture-forming units (CFU). Values are the mean of three independent experiments

The viable count of Cups fell below the detection limit after 5 days of incubation at 25 °C, while Cj did so at 6 days (Fig. 1A). In contrast, the culturabilities after incubation at 4 °C extended until 9 weeks for Cups and until 10 weeks for Cj (Fig. 1B). An environment contaminated with *Campylobacter*, particularly water, can pose a possible risk for transmission to animals and humans [9] and the present study showed that Cups retained its culturability in PBS under starvation stress and an aerobic atmosphere for several weeks at 4 °C. Indeed, in a case study of Cups infection in a hiker, the source of infection was suspected to be drinking of unsterilized spring water [10].

#### Survival of campylobacters under acidity, osmotic stress, and dryness

Cups showed a marked decline in the viable counts (~ 6 log_10_) after 2 days of incubation in 4.3 pH medium at 4 °C and was not detectable by 3 days. Cj was less severely affected and was culturable until 3 days of incubation (Fig. 1C). Cj was reported to grow well at pH 6.5–7.5, but its survival rapidly diminished at acidic pH [11]. The high sensitivity of Cups to acidity, suggesting lower survival under gastric acidity, may account for the lower incidence of Cups infection in humans. However, it has been suggested that ingestion of pathogenic *Campylobacter* with water [9] or certain foods [12], which act as buffers, may increase their survival in extremely acid conditions.

For osmolality stress, Cups survived for 9 days in high-osmolarity broth medium (4% NaCl) at 4 °C, but was more vulnerable than Cj (Fig. 1D). Under the same incubation conditions, Cj was culturable for 10 days with a ~ 3 log_10_ decline from the initial count. This agreed with a previous study that demonstrated a decrease of about 3 log_10_ in the Cj count within 14 days at 4 °C in the presence of 4.5% NaCl [13].

For dryness stress, the counts of both species declined simultaneously with continuous loss of moisture content in the inoculated pulp disks at 4 °C. Cups and Cj showed high sensitivity to dryness, and their viable counts fell below the detection limit by 6 and 9 days at 4 °C, respectively (Fig. 1E); this highlighted the importance of moisture content for survival of both species in the environment. This is in accord with previous reports that demonstrated long survival of Cj for more than 80 days in filter-sterilized stream water at low temperature [14] and a rapid decline in numbers under dry conditions [15]. Notably, both species failed to survive dryness, acidity and high osmolarity at 25 °C for 24 h, which highlight the key role of ambient temperature in survival of *Campylobacter* spp. to stresses in low nutrient environment.

#### Survival of *Campylobacter* species in dog feces

**Fig. 2:**
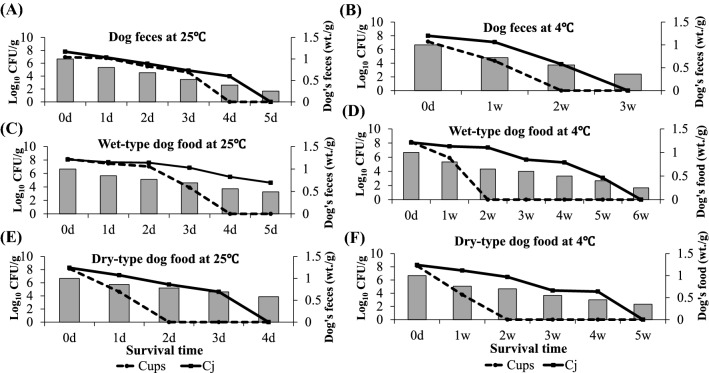
Survival of *Campylobacter* spp. in dog feces and dog food in aerobic atmosphere at different temperatures. **A** Survival of *C. upsaliensis* (Cups) and *C. jejuni* (Cj) in dog feces incubated at 25 °C. **B** Survival of Cups and Cj in dog feces incubated at 4 °C. **C** Survival of Cups and Cj in wet-type dog food incubated at 25 °C. **D** Survival of Cups and Cj in wet-type dog food at 4 °C. **E** Survival of Cups and Cj in dry-type dog food at 25 °C. **F** Survival of Cups and Cj in dry-type dog food at 4 °C. Survival capability was determined in terms of log_10_ culture-forming units (CFU). Values are the mean of three independent experiments *C. upsaliensis*

Cups survived in dog feces for about 3 days at 25 °C and for a longer time at low temperature (Fig. 2A, B). A marked difference in the survival of Cups relative to Cj was notable at the 1st week at 4 °C (~ 3 log_10_), suggesting that temperature may not be the sole factor influencing the survival of Cups in dog feces. Notably, the moisture content of the feces affected the survival of both of the *Campylobacter* spp. examined. A decline in the moisture content of dog feces was correlated with the decline in survival of both *Campylobacter* spp. (R = 0.8, *P* ˂ 0.01). The moisture content of animal feces has also been reported to affect the survival of *Campylobacter* spp. as well as that of other enteric bacteria such as *Salmonella* and *Escherichia coli* [16]. Previous reports have suggested that Cj survives for a variable time in cattle and poultry feces [17, 18]. However, there have been no available data on survival of *Campylobacter* spp. in dog feces.

#### Survival of *Campylobacter* species in dog food

In this study, we examined the survival of Cups and Cj in wet- and dry-type dog foods incubated aerobically at 25 °C and 4 °C. The viable count of Cups fell below the detection limit at 4 days after incubation at 25 °C (Fig. 2C) and at 2 weeks at 4 °C (Fig. 2D), while Cj was still culturable after 5 days at 25 °C (Fig. 2C) and after 5 weeks at 4 °C (Fig. 2D). The culturability of both *Campylobacter* spp. was shorter in the dried food than in the wet food (Fig. 2E, F). The survival of *Campylobacter* spp. in dog food (Fig. 2C–F), was correlated with its moisture content at both temperatures (R = 0.7–0.9, *P* ≤ 0.01). Many factors affect the survival of microorganisms in foods, including moisture, osmolarity and acidity [19]. The relatively short survival of Cups in dog food, especially dry-type food, may be attributed to sensitivity to these stressors. The present data on the survival of Cups in dog feces and dog food suggest an infectious source of transmission to other animals and to humans, although epidemiological data for dogs have been unavailable up to now.

*Campylobacter* spp. survive in the environment through mechanisms such as aerotolerance, biofilm formation, adaptive tolerance responses and transformation to a viable but nonculturable (VBNC) [6]. In all of the present experiments, the culturability of Cups was lower than that of Cj to a varying degree. This inter-species difference in survival tolerance to stress has been reported before [20]. One possible explanation for this is the variation in catalase production by these organisms. It is considered that catalase produced by *Campylobacter* spp. protects them from damage caused by oxygen radicals [21]. As Cups was originally described as a catalase-negative or weakly positive organism [22], this may account for its lower survival capability in comparison to Cj under aerobic conditions.

The risk arising through continuous and daily contact with household dogs, especially for children, has been emphasized. For prevention of new human cases, there is an urgent need for better public awareness and improved detection techniques for Cups. The present study provides the first information on the survival of Cups in the rearing environment of dogs, which may help to clarify possible routes of infection and prevent transmission of this pathogen to humans.

## Limitations

This study has some limitations. We have investigated only one type strain as a representative *Campylobacter* spp. and only two temperatures as an initial proof of concept. Furthermore, it is suggested that the phenotypic differences may be attributed to the high genetic diversity among *Campylobacter* spp. Therefore, the differences in survival patterns between Cups and Cj may be reflected in part by interspecies differences in stress response genes. Although the whole genomes of some Cups strains have been determined, the key genes related to the survival strategies of Cj have not yet been fully examined. While this study limited our ability to assume causality, findings may inform the direction of future research to understand the survival abilities of campylobacters under environmental stresses.

## Data Availability

The datasets used and/or analysed during the current study available from the corresponding author on reasonable request during the current study.

## Abbreviations

Cups: *Campylobacter upsaliensis*
Cj: *Campylobacter jejuni*
BCCM: Belgian Co-ordinated Collections of Micro-organisms
PBS: Phosphate-buffered saline
OD: Optical density
CFU: Colony-forming-unit
HCl: Hydrochloric acid
NaCl: Sodium chloride
VBNC: Viable but nonculturable
